# Physical Activity and Food Environments in and around Schools: A Case Study in Regional North-West Tasmania

**DOI:** 10.3390/ijerph19106238

**Published:** 2022-05-20

**Authors:** Sisitha Jayasinghe, Emily J. Flies, Robert Soward, Dave Kendal, Michelle Kilpatrick, Verity Cleland, Rebecca Roberts, Fadhillah Norzahari, Melanie Davern, Timothy P. Holloway, Sandra Murray, Kira A. E. Patterson, Kiran D. K. Ahuja, Roger Hughes, Nuala M. Byrne, Andrew P. Hills

**Affiliations:** 1 College of Health and Medicine, University of Tasmania, Hobart, TAS 7250, Australia; robert.soward@utas.edu.au (R.S.); michelle.kilpatrick@utas.edu.au (M.K.); verity.cleland@utas.edu.au (V.C.); timothy.holloway@utas.edu.au (T.P.H.); sandra.murray@utas.edu.au (S.M.); kiran.ahuja@utas.edu.au (K.D.K.A.); roger.hughes@utas.edu.au (R.H.); nuala.byrne@utas.edu.au (N.M.B.); andrew.hills@utas.edu.au (A.P.H.); 2 School of Natural Sciences, University of Tasmania, Hobart, TAS 7000, Australia; emily.flies@utas.edu.au; 3 Healthy Landscapes Research Group, University of Tasmania, Hobart, TAS 7000, Australia; dave.kendal@utas.edu.au; 4 School of Geography, Planning and Spatial Sciences, University of Tasmania, Hobart, TAS 7000, Australia; 5 Menzies Institute for Medical Research, University of Tasmania, Hobart, TAS 7000, Australia; 6 Australian Urban Observatory, Centre of Urban Research, RMIT University, Melbourne, VIC 3000, Australia; rebecca.roberts@rmit.edu.au (R.R.); fadhillah.norzahari@rmit.edu.au (F.N.); melanie.davern@rmit.edu.au (M.D.); 7 Centre for Health Equity, Melbourne School of Population and Global Health, University of Melbourne, Melbourne, VIC 3000, Australia; 8 College of Arts, Law and Education, University of Tasmania, Hobart, TAS 7250, Australia; kira.patterson@utas.edu.au

**Keywords:** childhood obesity, physical activity, food environment, spatial analysis, NW Tasmania, regional Australia, schools

## Abstract

A better understanding of the physical activity (PA) infrastructure in schools, the walkability of neighborhoods close to schools, and the food environments around schools, particularly in rural, socioeconomically challenged areas such as the North-West (NW) of Tasmania, could be important in the wider effort to improve the health of school-age children. Accordingly, this research aimed to assess PA resources, walkability, and food environments in and around schools in three socioeconomically disadvantaged, regional/rural Local Government Areas (LGAs) of Tasmania, Australia. A census of schools (including assessment of the PA infrastructure quality within school grounds), a walkability assessment, and a census of food outlets surrounding schools (through geospatial mapping) were executed. Most of the schools in the study region had access to an oval, basketball/volleyball/netball court, and free-standing exercise equipment. In all instances (i.e., regardless of school type), the quality of the available infrastructure was substantially higher than the number of incivilities observed. Most schools also had good (i.e., within the first four deciles) walkability. Numerous food outlets were within the walking zones of all schools in the study region, with an abundance of food outlets that predominantly sold processed unhealthy food.

## 1. Introduction

Physical inactivity and poor dietary habits coincide with a rapid increase in the prevalence of childhood overweight, obesity, and associated metabolic diseases [1,2]. There is a well-established link between habitual physical activity (PA), ideally commencing in the early years of life (e.g., toddlers, preschoolers, children, adolescents, etc.), and a healthy, productive life [3]. For example, investment in PA participation in early life is efficacious for the improvement of physical health (e.g., motor skills), social health, and psychological and cognitive health [4,5,6,7]. Good dietary habits are also significant determinants of the health status of children [8,9]. Schools play a central role in the provision of opportunities for children and adolescents to be physically active and to develop good dietary habits [10], given that children and youth spend a significant proportion of their time (roughly six hours per day for at least 75% of the year) at school [11].

Environmental factors are increasingly recognized as important determinants of health outcomes, and the environment in and around schools likely shapes health outcomes for children and youth. Within schools, reduced access to PA infrastructure and lack of time allocated for PA are the most common barriers to participation in developed economies such as Australia, the United States of America, and the United Kingdom [12,13,14,15]. Dietary habits are influenced by food environments, which in turn are shaped by local economic, policy, and sociocultural contexts. Although inconclusive, there is an expanding body of evidence on how the retail food environment surrounding schools can impact food consumption patterns and children’s body weight [16]. Some evidence suggests a positive association between increased accessibility to food outlets with unhealthy food items and overweight/obesity prevalence in school students [16,17].

A useful way of understanding local PA environments related to health outcomes is ‘walkability’—the extent to which the built environment is conducive to walking for recreation or transport. Walkability is a key contributor to PA and an overall healthy lifestyle [18,19,20]. Recent advancements in Geographic Information Systems data (e.g., raster and vector data) and methods (e.g., geocoding, buffer/network/overlay and spatial analysis) enable reasonably accurate forecasting of walkability in a variety of settings [21]. While associations between increased walkability and increased habitual PA in adults are reasonably well established [22,23,24,25,26], less is known about these relationships in children [27,28]. There is some evidence to suggest greater walkability around children’s homes relates to higher levels of moderate-to-vigorous physical activity (MVPA) [29]. However, a paucity of evidence exists regarding walkability in the vicinity of schools [30], and less is known about the food environment within ‘walkable’ areas around schools.

The social environment is also important. The makeup of obesogenic environments around schools is influenced by social strata, and schools in regions of lower socio-economic status (SES) are often surrounded by suboptimal opportunities for a healthy lifestyle, primarily driven by factors such as lower parental education, lower familial income, and negative peer pressure [30,31]. Consequently, this has contributed to a higher prevalence of childhood overweight and obesity in lower SES areas, with the gap between the top and bottom end of the SES spectrum continuing to widen in many developed economies [32,33,34]. Lack of adequate access to PA resources and healthy food is intrinsically linked with the SES of the community in which a school is located. Schools in lower SES areas may also face additional challenges incorporating PA into the school day, and consequently, children from these schools may be disproportionately inactive compared to children in more affluent neighborhoods [35,36,37,38]. Existing evidence indicates that deprivation amplification (the process by which individual or household deprivation (e.g., low income) is amplified by area level deprivation (e.g., sub-optimum PA and food infrastructure)) is also commonplace in local food environments in low-SES regions [39,40,41]. The combination of lower SES, physical inactivity, and unhealthy dietary habits increases the risk of overweight/obesity and poses a significant challenge for health and wellbeing in such communities.

Evidence from Australian adults suggests that less than 50% of the population meets the current recommendations for PA [42], with the prevalence of inactivity higher amongst individuals living in rural settings [43]. For instance, according to recent government reports, only 33.5% of Tasmanian children aged 2–17 years are meeting PA recommendations [44]. Rural Tasmania, including in the North-West (NW) of the state, has some of the highest rates of overweight and obesity and lowest levels of PA in the country [45]. The region is also challenged by limited access to healthy foods [46]. Nevertheless, a dearth of insights into food and PA environmental characteristics of schools currently exists in regional and rural areas of Australia. As such, research aimed at rectifying this gap using multipronged assessment techniques (e.g., systematic observational audits and spatial analysis) is of the highest priority.

Elements of both PA and food environments in and around schools are vital components in the overall ‘obesogenic environment’—the net result of biological, behavioral, and environmental influences that have an impact on the fat mass of individuals and populations by acting through the mediators of energy intake and expenditure [47]. A better understanding of the PA infrastructure in schools, the walkability of school neighborhoods, and the food environments around schools could be important in the wider effort to improve the health of school-age children. The aims of this research, in three socioeconomically disadvantaged NW Tasmanian towns, were to (1) assess the type, quantity, features, amenities, and quality of PA resources within schools, and (2) characterize the walkability and food environments within designated buffer zones around schools.

## 2. Materials and Methods

### 2.1. Study Design

This case study included a census of schools (including assessment of the PA infrastructure quality within school grounds), a walkability assessment, and a census of food outlets surrounding schools in three purposefully selected Local Government Areas (LGAs) in NW Tasmania: Burnie, Devonport, and Circular Head. The selection of these regional LGAs was consistent with the sentinel sites chosen for a larger obesity prevention project in the region. Regional Australia includes all the towns, small cities, and areas that lie beyond the major capital cities (Sydney, Melbourne, Brisbane, Perth, Adelaide, and Canberra) [28]. An LGA is an administrative division with responsibility vested in local government (Local Government Act 1993). In brief, the selected LGAs are classified as Remoteness Area 2 (Inner Regional Australia) and 3 (Outer Regional Australia), according to the Australian Statistical Geography Standard classification system.

### 2.2. Sampling of Schools

A comprehensive list of schools within each of the three LGAs was generated using a stepwise approach. First, a list of all government and non-government schools (including Catholic or Christian schools) was generated using online searches of the Tasmanian State Government, Tasmania Private Schools, and the Independent Schools Tasmania web pages. Lists were then cross referenced and verified via Google maps, the Australian Curriculum, Assessment and Reporting Authority website (https://www.acara.edu.au/, accessed on 1 June 2021), and in-person communication with Business Managers and Principals of each of the schools. A total of 36 schools from Devonport (*n* = 14), Burnie (*n* = 15), and Circular Head (*n* = 7) were included in the study. Direct access to schools was granted by Principals and (or) business managers as needed.

### 2.3. Physical Activity Environment

#### 2.3.1. Quality Assessment of PA Resources

The quality of the PA infrastructure in all schools was assessed using the Physical Activity Resource Assessment (PARA) instrument [48]. Each infrastructure item within the school premises was coded for quality using a three-category quantitative system as 3—good, 2—mediocre, or 1—poor, in accordance with the standards of quality prescribed in the PARA instrument. Additionally, each piece was rated on overall incivilities (i.e., elements that would decrease the satisfaction associated with usage of the infrastructure). Incivilities were rated as 3—high, 2—medium, or 1—low. Overall, 37 components (13 features, 12 amenities, and 12 incivilities) for each school were assessed and rated by trained researchers. Reliability of inter-rater scores was checked using Cronbach’s alpha values for all three sites. For clarity in data presentation, a composite quality score (QS) is presented as opposed to individual feature and amenity scores (i.e., QS = mean (feature score + amenity score)). Private and public schools from all sites were classified as Primary (from Kindergarten to grade 6), Secondary (from grade 7 to 12), and other (from Kindergarten to grade 12).

#### 2.3.2. Walkability Index

Walkability was calculated as the total length of high and low traffic roads (see below for classification) around schools.

Low traffic roads:

- Pedestrian thoroughfare
- Local road
- Access roads
- Undetermined roads

High traffic roads:

- Collector road
- Sub-arterial road
- Arterial road
- National or state highway

The maximum walkable distance around a primary school was set to 800 m and the walkable distance around a secondary school set to 1600 m (a street network buffer that could be walked briskly between 5 and 15 min), based on previously published research works [49]. ArcGIS 10.5.1 with Network Analyst extension—a commonly used online cloud-based mapping and analysis technique—was used to identify all pedestrian-accessible roads around each school and assign the road classification/road type to each road segment. The following steps were undertaken sequentially:

1. Create a network dataset from the Public Sector Mapping Agency (PSMA) roads;
2. Create a service area layer using the network data set;
3. Solve for 800 and 1600 m distances;
4. Spatially join the attribute of the input PSMA roads to the Network Analyst output;
5. Calculate total road lengths by road type;
6. Map output data (see below for details).

Deciles of school walkability were created by dividing the length of all high-traffic roads within the specified distance (i.e., 800 m or 1600 m) around a school by the total length of all walkable roads around a school and multiplying by 100 to express it as a percentage.

### 2.4. Food Environment

A comprehensive list of food outlets around schools were initially identified through business registration lists (obtained via LGA Environmental Health Officers) for each of the LGAs. These were subsequently cross referenced and confirmed through online verification by trained research personnel. Overall, 10 categories of food outlets were identified, as outlined below.

- Restaurant—Seated venue where food is purchased and primarily eaten onsite.
- Canteen—Where food is prepared/served and associated with a school, aged care, or sporting facility site.
- Take Away—Where food is prepared and purchased to take away.
- Fruit and Vegetable Market—Primarily sells fruit and vegetables.
- Supermarket—A primarily self-service shop selling foods and household goods.
- Manufacturer/distributor—Manufactures or processes food that is mainly sold on to other businesses for resale (could be home-based or larger commercial operation).
- Bakery—Produces baked goods/bakery products.
- Catering—Mobile business that provides prepared food (e.g., food vans, caterers that cater for events, service/special interest clubs, etc.).
- Specialty food store—Butcher or fishmonger.
- Fast Food/Franchise—Business belonging to a franchise and sells fast food primarily to take away.

For analytic purposes, food outlets were stratified into ‘Tier 1’ and ‘Tier 2’ outlets using previously published approaches [50,51]. In brief, ‘Tier 1’ food outlets included green grocers, butchers, supermarkets, and health food shops while ‘Tier 2’ outlets included fast food outlets (chain and non-chain), bakeries, sweet food retailers, and convenience stores. Visibility of fruit and vegetables in the outlet consumer view-space (as judged by research team members) and the level of ‘food processing’ (defined as all methods and techniques utilized by food, drink, and associated industries to convert fresh foods into food products) were used as criteria for this dichotomy. Using QGIS (version 3.18; [52]), an open source Geographic Information System that enables the utilization of numerous geospatial vector and raster file types and database formats, 800 m or 1600 m buffers were created around all primary or secondary schools, respectively, and all Tier 1 and Tier 2 outlets within these buffers was calculated using the ‘count points within polygons’ tool. For the purpose of this assessment, K–12 schools were considered to be primary schools to give a more conservative estimate of accessible food outlets.

## 3. Results

### 3.1. Variety and Quality of PA Infrastructure in Schools

Demographic characteristics of the study area and schools are presented in Table 1. The SES of the three study regions is below the national average for Index of Relative Socio-economic Advantage and Disadvantage (IRSAD). Circular Head, the least populous area, had seven schools, with an average student body of 165 pupils. Burnie and Devonport—twice as populated as Circular Head—had 15 and 14 schools, respectively. Most of the schools were public in all three study sites. On average, each school had more than 13 elements (i.e., features and amenities) that supported PA opportunities.

**Figure 1:**
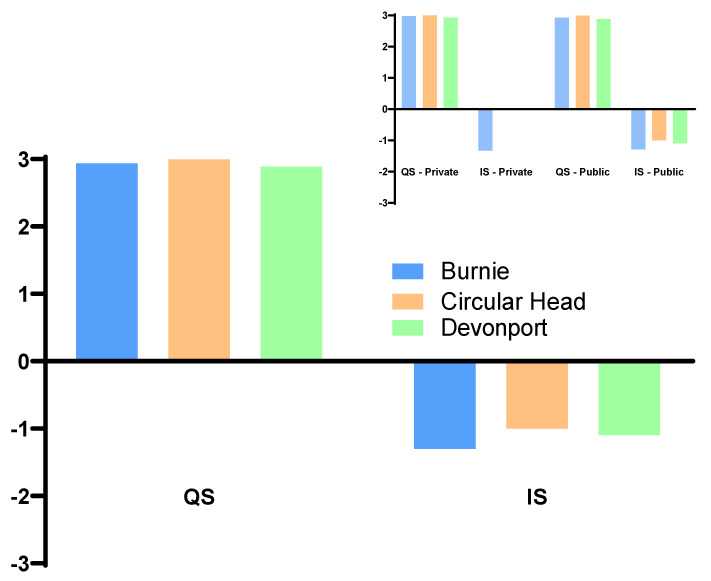
Quality rating for physical activity infrastructure of all schools (main figure) and public and private schools (inset) in Burnie, Devonport, and Circular Head. Inter-rater reliability Cronbach’s alpha values were 0.991, 0.935, and 0.982 for Devonport, Circular Head, and Burnie, respectively. ‘Quality’ of infrastructure was objectified as follows: 3—good, 2—mediocre, or 1—poor; ‘Incivility’ associated with infrastructure was objectified as follows: 3—high, 2—medium, or 1—low.

Most of the schools in the study region had access to an oval, basketball/volleyball/netball court, and free-standing exercise equipment (Table 2). In contrast, designated soccer pitches, tennis courts, and swimming pools were not available inside most school premises. In all instances (i.e., regardless of school type), the quality of the available infrastructure was substantially higher than the number of incivilities observed. No incivilities were recorded within private schools in both Devonport and Circular Head (Figure 1).

### 3.2. Walkability around Schools

**Figure 2:**
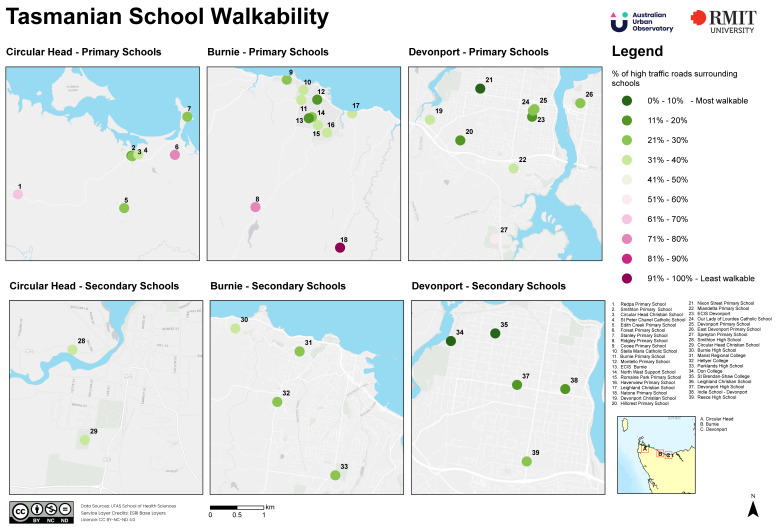
Walkability around primary and secondary schools.

Walkability around schools in the study region is depicted in Figure 2. The majority of schools had good (i.e., within the first four deciles) walkability. Since walkability for K–12 schools was calculated at both 800 and 1600 m, different walkability scores (Leighland Christian School 4th and 6th decile at 800 and 1600 m, Circular Head Christian School 2nd and 4th decile at 800 and 1600 m) were indicated for each distance class (Figure 2). Schools in Devonport appeared to be the most walkable, with no school reported in deciles 7–10. In contrast, Burnie and Circular Head contained a more schools with surroundings that were not conducive (deciles 8–10) to walking (Figure 2).

### 3.3. Food Environment

**Figure 3:**
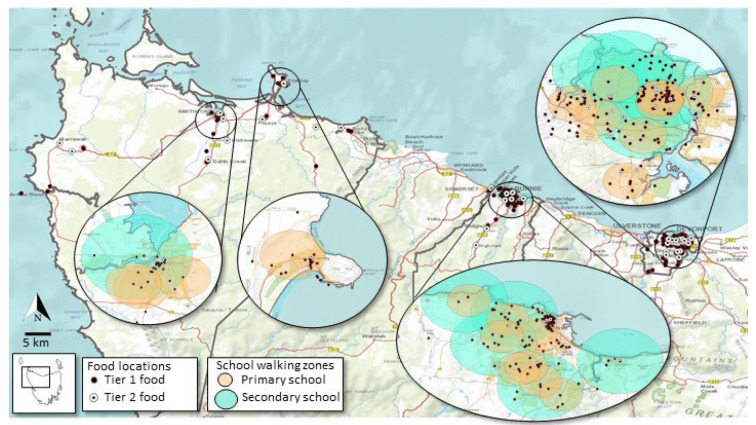
Access to Tier 1 and Tier 2 food outlets.

Numerous food outlets were within the walking zones of all schools in the study region (Figure 3). An overwhelmingly large number of these outlets were Tier 2, which predominantly sell processed unhealthy food (Table 3). Primary schools in the Burnie LGA had the highest Tier 2:Tier 1 ratio (15.83), followed by Circular Head (10.00) and then Devonport (9.03). Burnie also had the highest Tier 2:Tier 1 ratio (16.10) for secondary schools (Table 3). Overall, secondary schools in Circular Head had the lowest Tier 2:Tier 1 ratio (5.67) of all study sites (Table 3).

## 4. Discussion

This research aimed to assess PA resources, walkability, and food environments in and around schools in a socioeconomically disadvantaged, regional/rural area of Tasmania, Australia. Overall, most schools in the three LGAs had high-quality PA infrastructure within school premises, were located within walkable surroundings, but were proximate to an overwhelming majority of Tier 2 (unhealthy) food outlets.

Lower SES is often associated with lack of appropriate facilities and consequent inactivity in youth [53,54]. As such, for a region with relatively low-SES, it is gratifying to see such variety and quality in the available PA infrastructure (i.e., a high degree of playability). Nevertheless, whether availability and quality of PAI within schools is translating into habitual PA is questionable, with current public health statistics indicating that only 33.5% of Tasmanian children aged 2–17 years are meeting PA recommendations [44]. Given that schools provide a unique setting to foster positive PA habits that have the potential to last for decades [11,55], it is paramount that all possible efforts are undertaken to optimize the use of these facilities. Initiatives such as Healthy Tasmania and Getting Australia Active III—with nuanced plans for increased local leadership and advocacy, multi-sectoral collaboration, knowledge sharing, infrastructure development, implementation monitoring and evaluation—constitute steps in the right direction.

Walkability scores for most schools in the study region demonstrated that the immediate surroundings are conducive to active modes of transportation such as walking and cycling. Despite being a vital contributor to children’s total PA [56], active commuting to schools has been constantly declining in a variety of settings in recent times [57,58,59]. In the Australian context, the rates of children walking to and from school has also been declining rapidly, particularly in low-SES areas [59]. Walkability measurements are reliant both on micro and macro environmental factors [60,61]. Given the neighborhood characteristic differences in urban and rural settings, a possibility exists that unique neighborhood walkability indices might be necessary for regions such as NW Tasmania [60,62]. For instance, the number of four-way intersections and higher residential density are positively linked with active commuting to schools in urban but not necessarily in rural settings [63,64,65]. In urban areas, a combination of proximity to school (ideally less than 2 km), elements of traffic (exposure, control, low speed, etc.), and extensive walking infrastructure (walking paths, sidewalks, etc.) has been found to increase the odds of active commute to school [66,67].

In rural settings, the walkability of school neighborhoods may be associated with poorer PA outcomes, as students are more likely to live outside the ‘walkable’ catchment compared with dense urban settings. The geographical locations of residential dwellings can be quite dispersed in regional towns, which results in an over reliance on motorized transport for essential commutes. With limited or no public transportation and long traveling distances, rural Australia’s dependence on private transportation is a good case in point [68]. As such, a favorable score on a walkability index may not necessarily translate to increased participation in active commuting unless living in close proximity to the school, and different walkability measures may be required in rural areas.

Furthermore, components other than the physical environment, such as attitudes of parents or caregivers and their perceived barriers on children’s free movement, are also primary correlates of whether a walkable environment translates into meaningful PA patterns in school-aged children [69,70]. For instance, Australian adults are highly conservative with regards to allowances made for ‘independent walking and cycling’, with most parents restricting their children’s independent movements to within 500 m of home [70,71,72], despite the well-known benefits of these activities [73]. Social and built environment factors and adult perceptions of child safety are also important mitigating factors of unsupervised PA in children [74,75,76]. Factors such as perceived ‘stranger danger’, traffic volume and speed, and bullying have been reported as contributors to adult concerns of child safety [77]. In addition, lower SES status and education level of parents—issues of concern in NW Tasmania—has also been reported to be associated with less independent mobility in children [70,78].

We observed an abundance of Tier 2 (unhealthy) food outlets surrounding all schools in the study region, akin to the ‘food swamps’—areas with abundant food outlets selling unhealthy quick serve food—described elsewhere [79,80]. To put this in context, secondary schools in Circular Head, which have the lowest unhealthy:healthy outlet ratio in our study, have five times as many unhealthy food stores than healthy food stores (Table 3). Although concerning, such characteristics are not uncommon in food retail environments in developed countries [81,82]. Of particular concern is the recent evidence suggesting that an increased diversity of contemporary foodscapes (e.g., grocery stores, gas stations, takeaways) around schools provides an exponential increase in the availability of ultra-processed, energy-dense products [83,84,85]. From a health perspective, there is a strong association between abundance of unhealthy retail food outlets (e.g., fast food and convenience stores) and consumption of poorly nutritious, energy-dense food by children and obesity [86,87]. There are multiple motivational factors (e.g., price, health, and convenience) alongside the choice of products within a store and choice of the store itself that are influencing the bi-directional relationship between diet cost and diet quality [88]. It is highly plausible that a significant proportion of the energy intake of NW Tasmanian children is coming from unhealthy sources. In the broader obesity context, more work needs to be done to assess the food choices in school neighborhoods and around food environment exposure along the routes to school. Existing evidence indicates that measurable changes in anthropometric indices (e.g., body mass index (BMI) and waist circumference, etc.) can be achieved with increased availability of healthy food options in the immediate vicinity (and en route to) school [89,90].

## 5. Conclusions

This study provides much needed evidence on the obesogenic environment in and around schools in NW Tasmania. The holistic approach undertaken in this instance (i.e., assessment of in-school PA infrastructure, walkability, and food environment together) provides a comprehensive snapshot of the environmental determinants of obesity in schools in NW Tasmania. The lack of consistent spatial data from regional jurisdictions has been a long-standing impediment to the progression of walkability research in Australia. As such, the state-of-the-art objective analysis of walkability around schools in regional NW Tasmania adds much needed information to existing national data. However, this research does not include an in-depth analysis of whether the assessed components of the school PA and food environments are manifesting as changes in PA participation and/or dietary choices of the children. Furthermore, the lack of pedestrian network data—an inherent limitation in all road network-based walkability calculations [91]—may limit the generalizability of our observations to other jurisdictions. Overall, this study observed that children in three NW Tasmanian LGAs have numerous PA facilities within schools, have reasonably ‘walkable’ neighborhoods around schools to support active commuting, but are surrounded by mostly unhealthy food outlets. Schools, particularly their sports facilities, provide an ideal platform for promoting PA and enabling positive ‘habit forming’, which can be beneficial for longer-term health and wellbeing. As such, specific programs targeted to promote the greater usage of this infrastructure should be endorsed. Government-imposed mandates in relation to availability of healthier foods might be necessary to tilt the balance back towards Tier 1 outlets. As indicated in the recent ‘Scorecard and Priority Recommendations for the Tasmanian Government report’, Tasmania has had extremely limited action in relation to supporting LGAs to decrease access to unhealthy foods through legislation, governance, monitoring, and funding in recent times. Creation of ‘healthy eating zones’ or ‘green food zones,’ and (or) bans on unhealthy food outlets around schools—which have been effective elsewhere [16,92]—could also be viable options. Overall, there has never been a more opportune moment to intervene in children’s PA levels, and future research should also investigate the possibilities and effectiveness of other popular forms of PA amongst children. Simultaneous investigations of walkability and cyclability could be a step in the right direction in this regard [73]. It may also be prudent for future research to focus on improving the technical aspects of walkability assessment (e.g., frequent utilization of three-dimensional pedestrian networks to improve accuracy), upgrading/standardizing food outlet classification (e.g., considering a variety of outlets, food items sold, and purchasing patterns), and the frequent utilization of objective measures of PA (e.g., accelerometry data [93]).

## Figures and Tables

**Table 1:** Characteristics of the study areas and schools.

	Characteristic	Burnie	Circular Head	Devonport
Area demographics	Population > 18 years *	14,308	5917	18,919
	Population < 18 years ^#^	5006	2234	6332
	Total population	19,314	8151	25,251
	Population density per km^2^	31.6	1.7	227.5
	Geographical area (km^2^)	611	4898	111
	SES status ^†^	886	896	915
School characteristics	Count	15	7	14
	Average enrolment (min/max)	372 (30/763)	165 (42/306)	389 (110/800)
	ICSEA standing ^++^	950 (885/1033)	915 (862/1002)	954 (872/1019)
	% Public/Private	80/20	71/29	79/21
	% Primary/Secondary/Other ^$^	67/27/7	72/14/14	64/29/7
PA infrastructure	Average features (min/max)	6.80 (2/9)	5.33 (4/6)	6.36 (5/8)
	Average amenities (min/max)	8.2 (6/10)	8.83 (7/10)	9.18 (7/11)

* Australian Bureau of Statistics 2016 Census; ^†^ IRSAD (Index of Relative Socio-economic Advantage and Disadvantage) (National average = 958, range: 400–1239); ^++^ ICSEA (Index of Community Socio-educational Advantage); ^$^ K–12; ^#^ The Health and Wellbeing of Tasmania’s Children and Young People Report.

**Table 2:** Available facilities stratified by school type and location.

		AFL	Basketball/Volleyball/Netball	Soccer	Tennis	Swimming	Play/Exercise Equipment
Devonport	Primary	6	7	6		1	7
	Secondary	4	4	3	2		4
	Other						
Circular head	Primary	4	4		1		4
	Secondary	1	1		1		1
	Other	1	1				1
Burnie	Primary	5	8	1	2	2	10
	Secondary	4	4	3	3		3
	Other		1	1			1
Total (% of schools)		25 (76%)	30 (91%)	14 (42%)	9 (27%)	3 (9%)	31 (94%)

**Table 3:** Abundance of Tier 2 (unhealthy) and Tier 1 (healthy) food outlets within walking distance of primary and secondary schools.

	Primary			Secondary		
Region	Unhealthy Count	Healthy Count	Ratio of Unhealthy to Healthy	Unhealthy Count	Healthy Count	Ratio of Unhealthy to Healthy
Burnie	95	6	16:1	161	10	16:1
Circular Head	40	4	10:1	34	6	6:1
Devonport	298	33	9:1	385	41	9:1

## Data Availability

Not applicable.
